# Modulation of the Cutaneous Silent Period in the Upper-Limb with Whole-Body Instability

**DOI:** 10.1371/journal.pone.0151520

**Published:** 2016-03-16

**Authors:** Nathanial R. Eckert, Brach Poston, Zachary A. Riley

**Affiliations:** 1 Department of Kinesiology, Indiana University, Bloomington, Indiana, United States of America; 2 Department of Kinesiology and Nutritional Sciences, University of Nevada-Las Vegas, Las Vegas, Nevada, United States of America; 3 Department of Kinesiology, Indiana University-Purdue University, Indianapolis, Indiana, United States of America; University of Ottawa, CANADA

## Abstract

The silent period induced by cutaneous electrical stimulation of the digits has been shown to be task-dependent, at least in the grasping muscles of the hand. However, it is unknown if the cutaneous silent period is adaptable throughout muscles of the entire upper limb, in particular when the task requirements are substantially altered. The purpose of the present study was to examine the characteristics of the cutaneous silent period in several upper limb muscles when introducing increased whole-body instability. The cutaneous silent period was evoked in 10 healthy individuals with electrical stimulation of digit II of the right hand when the subjects were seated, standing, or standing on a wobble board while maintaining a background elbow extension contraction with the triceps brachii of ~5% of maximal voluntary contraction (MVC) strength. The first excitatory response (E1), first inhibitory response (CSP), and second excitatory response (E2) were quantified as the percent change from baseline and by their individual durations. The results showed that the level of CSP suppression was lessened (47.7 ± 7.7% to 33.8 ± 13.2% of baseline, *p* = 0.019) and the duration of the CSP inhibition decreased (*p* = 0.021) in the triceps brachii when comparing the seated and wobble board tasks. For the wobble board task the amount of cutaneous afferent inhibition of EMG activity in the triceps brachii decreased; which is proposed to be due to differential weighting of cutaneous feedback relative to the corticospinal drive, most likely due to presynaptic inhibition, to meet the demands of the unstable task.

## Introduction

A single pulse of high intensity electrical current delivered to the digits of the hand produces a period of decreased electromyographic (EMG) activity, or a cutaneous silent period (CSP), in muscles of the upper limb (e.g. thenar, triceps brachii) during voluntary contractions [1–3]. Converse to the nociceptive withdrawal response, which is an ‘excitatory strategy’ to activate primarily flexor muscles that were previously quiescent to withdraw the limb [4], the CSP is a protective mechanism using an ‘inhibitory strategy’ to suppress activity in muscles [5]. When noxious stimuli are transduced via relatively slow-conducting Aδ afferent fibers to the motorneuron pool through direct or indirect synaptic connections [3, 6, 7], an inhibitory spinal reflex response results that can aid in releasing a grasp or halting forward reaching movements [8, 9].

A critical feature of the CSP response is that it is mutable to the task, a fundamental requirement for protecting the upper limb when interacting with dynamic environments [3, 9, 10]. Presumably, adaptability in the CSP is possible due to the wide distribution of the small-diameter fiber inputs to the motorneuron pools of several muscles [11]. Consequently, the cutaneous receptive fields activated by the stimulus, and the afferent fibers carrying the activation signal, aid in the coordination of activity in one or more muscles best suited to protect the area of tissue from the noxious stimuli [12, 13]. This is referred to as the modular organization of nociceptive reflexes, and the basic theory can be applicable whether the end result is excitation or inhibition of a given muscle (e.g. excite flexor, inhibit extensor). This mutability has previously been demonstrated as Kofler [5] demonstrated that the CSP differs in timing and magnitude of inhibition depending on the intensity of stimulation across hand muscles. The intensity-dependent change in the CSP was suggested to allow for precise adjustments, or a more discerning response to cutaneous input versus a standard all-or-none response. Within a dynamic environment sensory input arrives from a variety of sources (i.e. peripheral and supraspinal sources) needing to be processed in the most appropriate manner dictated by the current state of the system. Optimal feedback control theory (Todorov and Jordan, 2002) provides a mechanism by which the ability to adjust features of the CSP (magnitude of response, coordination of activity, etc.) to allow for the maintenance of performance during a given task, while ignoring other sources of variability. The evidence presented further supports the function of the CSP as an adaptable spinal inhibitory reflex that can help protect the hand or limb from harm.

Though it is clear that the CSP provides a mechanism for regulating interactions in an ever-changing environment with the upper-limb, the organization of this reflex remains unknown. For example, it is unknown if the CSP is purely a localized spinal response, or if it is dependent on the state or demands on the rest of the body. Specifically, it is not clear if the CSP is subservient to other sources of sensory feedback and descending drive, or if the protective CSP mechanism takes precedence in the hierarchy of upper-limb motor control. There is data showing that when a motor evoked potential (MEP) is evoked during a CSP the MEP is suppressed, but not totally absent [14, 15]. This becomes difficult to interpret because the presence of the MEP during the CSP (even if suppressed) suggests the cortico-motorneuronal connections cannot be inhibited completely, yet the modulation of the MEP indicates a clear synaptic influence from the CSP spinal circuitry.

In order to approach this problem, the current study sought to investigate the characteristics of the CSP during a more functionally relevant task (i.e. whole-body instability). To date, a large portion of studies looking at the CSP have focused mainly on gripping tasks while isolating the upper-limb and forearm. However, conceptually these restraints are not present within everyday interactions requiring complex movements that necessitate greater control (i.e. the need to maintain balance). Therefore, in the current study, individuals grasped a rigid handle and received stimulation to the index finger while seated, standing on a stable surface, and standing on an unstable wobble board. It was hypothesized that if the CSP was a localized response then a similar response should be observed in all three conditions. However, if the CSP is influenced by the requirement to maintain whole-body balance then it was expected that the suppression of muscle activity would be significantly reduced to enable greater voluntary control of balance. Evidence of this modification would provide support for the functional relevance of the CSP while also providing information about the sensory-motor interactions between lower and upper limbs.

## Materials and Methods

### Participants

Ten healthy adults (8 males: 29.9 ± 7.5 yrs; range: 21–42 yrs) participated in the study. None of the subjects reported any neurological disorders or other upper limb musculoskeletal impairments. Each subject provided a written informed consent prior to participating. The protocol was approved by the Indiana University Institutional Review Board and was performed in accordance with the Declaration of Helsinki.

### Experimental Setup

CSPs were examined during three different conditions while the subjects sustained a low-intensity isometric elbow extension contraction with the right upper limb by extending the elbow and pushing against a vertical handle. Surface EMG activity was recorded during all of the experimental conditions from the abductor pollicis brevis (APB), abductor digiti minimi (ADM), biceps brachii long head (BIC), triceps brachii lateral head (TRI), flexor carpai radialis (FCR), extensor carpi radialis (ECR), anterior deltoid (AD), and posterior deltoid (PD) muscles in the right upper limb. Surface EMG signals were recorded with single differential bar electrodes (Delsys Inc, MA, USA). The skin overlying each muscle was cleaned prior to affixing the electrode over the individual muscle belly, parallel with the orientation of the respective muscle’s fibers. All of the EMG signals were sampled at a frequency of 2,000 Hz. The signals were amplified and conditioned using a 16-channel Bagnoli EMG System (Delsys) with high- and low-pass cut-off frequencies of 20Hz and 500Hz, respectively, before being stored at a final gain of 1,000x with Spike2 software (CED, Cambridge, UK). A single common ground electrode was placed over the acromion process of the right side of the body.

### Experimental Conditions

Subjects were first instructed to produce three maximal voluntary contractions (MVC) in which the single MVC trial with the highest extension force was used to set the level of triceps brachii EMG activity for the experimental trials. MVC trials were performed while the subject stood on a wobble board, so that they could not push with other muscles without risking becoming more unstable. Subjects were instructed to extend the elbow as hard as possible while maintaining upright posture and not leaning into the handle. Following the MVC trials the maximum triceps brachii EMG value was recorded and a horizontal line was set on a monitor in front of the subject that represented 5% of that maximum. Subjects were instructed to match that line with their triceps brachii EMG activity during each of the experimental trials by extending the elbow and performing an isometric contraction. In order to make the visual feedback display easier to follow the MVC values and 5% EMG matching line were rectified and smoothed over a 200ms window.

**Fig 1:**
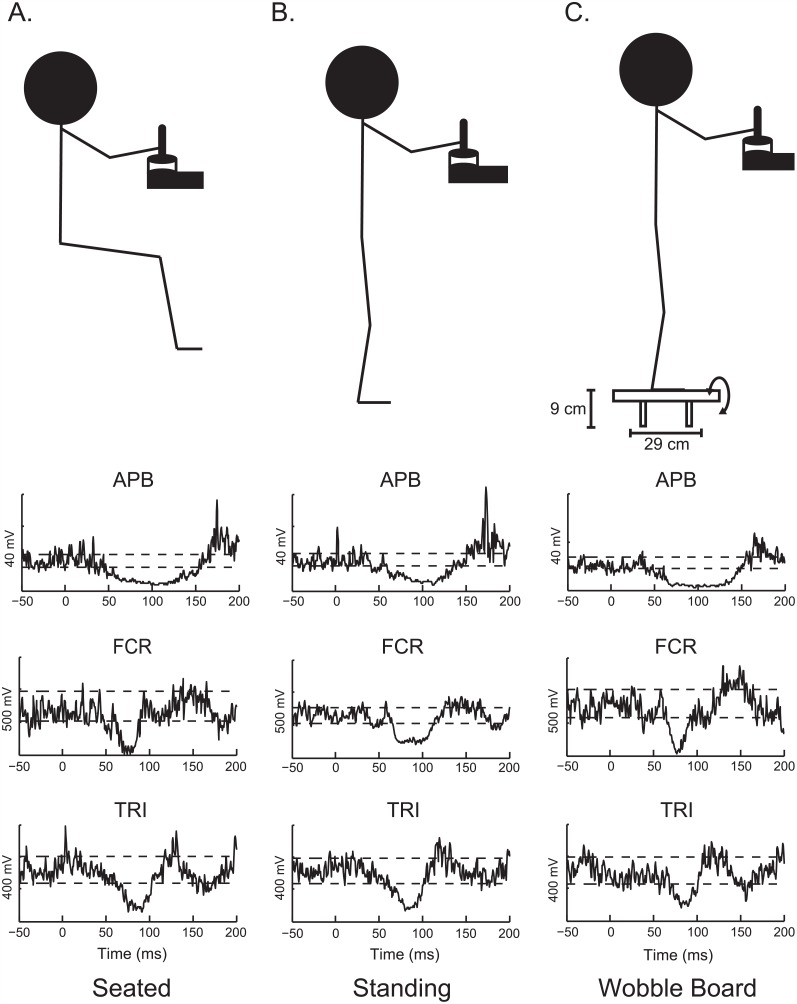
Representative data displaying CSP inhibition. A-C) Schematic showing the body configuration for each of the conditions (seated, standing and wobble board; respectively). Below each of the schematics depicting the body position are representative data from three upper limb muscles: abductor pollicis brevis (APB), flexor carpi radialis (FCR), and triceps brachii (TRI). The data show the average of 50 stimulation trials from 50 ms prior to the digit stimulation up to 200 ms following the digit stimulation. The horizontal dashed lines are plotted to show 80% and 120% of the baseline, background EMG that was used as a threshold for determining responses.

Each subject was tested in three conditions that were performed in random order. The conditions consisted of being seated, standing on a stable surface, and standing on a wobble board (unstable in the medial-lateral direction) while grasping a vertical handle with the right upper-limb (Fig 1). The three conditions introduced different levels of stability (more stable—less stable, e.g. seated—standing on wobble board). In the seated condition, the subject was confined to a position where the torso was upright and supported against a backrest. The second condition required the subject to stand upright with their feet 20 cm apart and parallel with each other while trying not to move. The last condition required the subject to stand on 9 cm tall wobble board with feet 20 cm apart and in line with each other while again trying to remain stationary as the board was free to roll from side-to-side. Shoulder and elbow flexion was kept as constant as possible (~45° and ~90°, respectively). The subjects were monitored to ensure that they remained upright and did not lean forward while pushing against the handle. Each subject performed the three conditions in the same testing session with 5 minutes of rest provided between trials.

### Stimulation Parameters

Noxious electrical stimulation (square-wave pulses, 0.5ms duration) was delivered to digit II of the right hand with a Digitimer DS7AH constant current electrical stimulator (Digitimer LTD, England, UK). The use of Digit II stimulation fell in line with previous investigations on the CSP providing the ability to compare previously limited interpretations with this model of testing [2, 5, 16]. Additionally, electrical stimulation of digit II activates the median nerve, which supplies cutaneous afferent information to all the muscles of interest. Sensory or perceptual threshold was determined by slowly increasing the electrical current until perceivable by the subject. Five stimuli were randomly delivered and the subject had to be able to detect all five to ensure the accuracy of perceptual threshold. The level of stimulus intensity utilized during the experiment was set at 10x perceptual threshold. This resulted in a stimulus intensity of between 30–50 mA for all subjects, which was consistent with other studies in the upper limb [5, 17]. Fifty total stimulations were delivered randomly (0.5Hz ± 0.2Hz stimulation rate) during the isometric contraction in each condition.

### Data Analysis

**Fig 2:**
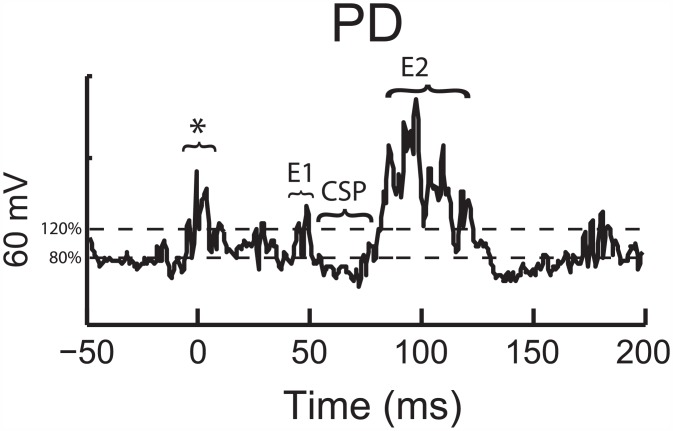
Representative data displaying reflex phases. Representative data from the PD demonstrating the identification of the different phases (E1, CSP, E2) within the cutaneous silent period as well as the EMG baseline data used to calculate the 80% and 120% thresholds. The * denotes the EMG period contaminated with stimulus artifact.

Each of the EMG signals were processed and the reflex responses were identified with custom programs written in MATLAB (Mathworks, MA, USA) by first removing the DC offset, band-pass filtering the signals at 20–500 Hz, then rectifying the signals. The series of 50 stimuli were averaged and superimposed to ensure reproducibility when identifying the phases of the reflex response. Individual inhibitory (cutaneous silent period, CSPs) and excitatory (E1 and E2, see Fig 2) phases were identified within each reflex response [18]. The nomenclature of CSP was used instead of other abbreviations (e.g. I1) to be consistent with other research in this area. The typical pattern of EMG activity comprising the reflex response was as follows: E1, CSP, and E2. Initially, the mean EMG amplitude during a 100 ms pre-stimulus baseline period (-100ms—Stimulation) was quantified and the onset of the excitatory responses (E1, E2) was determined by visual inspection when the EMG activity exceeded 120% of that baseline EMG value, signifying muscle activation (i.e. 20% increase in activity from average baseline EMG), following stimulation. On the other hand, the offset, or “ending” of the excitatory periods were determined visually as the point when the EMG activity dropped below this level (120% of baseline signifying a return to average baseline EMG), only if occurring more than 5 ms after the onset. The CSP period was then identified by visual inspection when the EMG activity fell below 80% of the baseline EMG activity (onset), signifying muscle inhibition, and back above 80% (offset) as long as the duration of the CSP was longer than 5 ms. These methods of reflex phase determination were utilized as they fall in line with previous work on cutaneous reflexes and demonstrate clear facilitatory and inhibitory effects[2, 5]. The time points for the onsets and offsets for each phase of the reflex response were recorded and then from that the individual phase durations were calculated. In addition, the % of inhibition (CSP) or excitation (E1, E2) was calculated by dividing the level of EMG activity during the individual phase (CSP, E1, or E2) by the baseline EMG amplitude [5, 17]. There were a few instances where either the E1 period or E2 period could not be accurately identified in a given muscle for a subject. However, the responses were identifiable in a minimum of 7 out of 10 subjects, and the CSP was identifiable in all muscles.

### Statistical Analysis

Statistical analyses were performed utilizing the statistics toolbox in MATLAB (Mathworks, MA, USA). Kruskal-Wallis non-parametric statistical tests were used to compare the percent of inhibition/ excitation across the three stability conditions (seated, standing, wobble board). A separate test was used for each of the three different phases of the reflex response (E1, CSP, E2). The same tests were used to examine the durations of the individual responses (E1, CSP, E2) across the stability conditions for each muscle. Multiple comparisons were then used to determine individual differences. Results were considered significant at *p <* 0.05. Results are presented in the text with the mean ± standard deviation and in the figures as the mean ± standard error of the mean (S.E.M.).

## Results

Representative EMG traces for a series of 50 stimuli that were averaged in a single subject are displayed at the bottom of Fig 1 for a time window of 50 ms pre-stimulus onset to 200 ms after the stimulus onset. Prevalence and latencies for both onsets and offsets for each reflex phase and condition are presented in Tables 1–3. Reflex habituation was identified via paired samples t-test of the average first CSP response with the last. Results demonstrated no habituation within the ADM (t(9) = 2.202, *p* = .055), APB (t(9) = -1.231, *p* = .250), ECR (t(9) = .447, *p* = .666), FCR (t(9) = -.244, *p* = .813), and TRI (t(9) = 1.948, *p* = .083), which falls in line with previous investigations[19]. In contrast, habituation was found within the Adelt (t(7) = 6.038, *p* = .001), Pdelt (t(6) = 7.120, *p* = .000), and BIC (t(6) = 6.055, *p* = .001) muscles.

**Table 1:** Prevalence/Latencies for each the E1 reflex phase across conditions.

E1								
**Seated**								
	**APB(10)**	**ADM(9)**	**BIC(7)**	**TRI(10)**	**FCR(7)**	**ECR(10)**	**AD(8)**	**PD(8)**
**Onset**	**30.9±4.2**	**38.2±6.2**	**27.6±10.8**	**18.0±6.2**	**28.5±7.9**	**32.5±6.9**	**43.5±10.3**	**23.1±10.9**
**Offset**	**39.8±4.9**	**57.4±10.2**	**43.5±13.8**	**24.1±7.3**	**43.1±12.3**	**39.5±8.9**	**62.8±15.8**	**37.2±17.9**
**Standing**								
	**APB(10)**	**ADM(10)**	**BIC(10)**	**TRI(10)**	**FCR(6)**	**ECR(10)**	**AD(8)**	**PD(9)**
**Onset**	**24.8±5.2**	**50.2±7.4**	**29.7±5.9**	**20.5±5.8**	**39.3±8.4**	**44.2±5.6**	**45.9±10.1**	**31.0±5.7**
**Offse**	**32.3±6.0**	**72.7±9.4**	**44.9±8.7**	**26.1±8.0**	**51.6±10.7**	**55.7±7.0**	**60.8±14.6**	**46.3±10.7**
**Wobble**								
	**APB(10)**	**ADM(10)**	**BIC(10)**	**TRI(10)**	**FCR(9)**	**ECR(10)**	**AD(8)**	**PD(9)**
**Onset**	**34.9±4.8**	**55.2±4.3**	**46.4±8.8**	**23.9±9.0**	**38.8±11.2**	**44.8±5.9**	**37.9±11.4**	**37.0±9.4**
**Offset**	**41.0±5.1**	**77.2±6.2**	**65.0±13.0**	**29.1±11.4**	**33.6±9.2**	**57.2±8.0**	**52.3±14.5**	**48.5±13.2**

Reflex prevalence is denoted within parentheses per condition and muscle. Latencies are presented as an average ± standard error of the mean in milliseconds.

**Table 2:** Prevalence/Latencies for each the CSP reflex phase across conditions.

CSP								
**Seated**								
	**APB(10)**	**ADM(9)**	**BIC(7)**	**TRI(10)**	**FCR(7)**	**ECR(10)**	**AD(8)**	**PD(8)**
**Onset**	**50.9±5.8**	**66.1±11.1**	**59.4±12.4**	**53.9±4.7**	**63.1±12.3**	**68.4±5.7**	**82.1±26.2**	**55.2±14.9**
**Offset**	**116.3±15.3**	**84.3±15.0**	**92.1±18.5**	**109.3±3.2**	**93.6±16.9**	**115.2±4.0**	**105.0±25.0**	**76.8±20.2**
**Standing**								
	**APB(10)**	**ADM(10)**	**BIC(10)**	**TRI(9)**	**FCR(6)**	**ECR(10)**	**AD(8)**	**PD(9)**
**Onset**	**58.1±5.2**	**87.9±7.4**	**71.2±5.9**	**63.3±5.8**	**60.8±8.4**	**72.2±5.6**	**67.0±10.1**	**70.8±5.7**
**Offset**	**126.8±6.0**	**123.7±9.4**	**103.1±8.7**	**105.0±8.0**	**82.6±10.7**	**120.6±7.0**	**84.9±14.6**	**88.1±10.7**
**Wobble**								
	**APB(10)**	**ADM(10)**	**BIC(10)**	**TRI(10)**	**FCR(9)**	**ECR(10)**	**AD(8)**	**PD(9)**
**Onset**	**61.3±4.8**	**77.0±4.3**	**85.4±8.8**	**49.2±9.0**	**77.2±11.2**	**77.5±5.9**	**60.1±11.4**	**50.6±9.4**
**Offset**	**121.7±5.1**	**124.2±6.2**	**111.7±13.0**	**82.1±11.4**	**99.9±9.2**	**118.8±8.0**	**82.1±14.5**	**68.3±13.2**

Reflex prevalence is denoted within parentheses per condition and muscle. Latencies are presented as an average ± standard error of the mean in milliseconds.

**Table 3:** Prevalence/Latencies for each the E2 reflex phase across conditions.

C) E2								
**Seated**								
	**APB(10)**	**ADM(9)**	**BIC(7)**	**TRI(10)**	**FCR(7)**	**ECR(10)**	**AD(8)**	**PD(8)**
**Onset**	**141.9±10.1**	**103.42±15.5**	**121.8±11.7**	**119.0±3.6**	**129.7±7.5**	**126.0±4.1**	**82.2±18.7**	**130.7±20**
**Offset**	**201.4±15.7**	**129.9±16.3**	**170.8±8.0**	**157.0±6.3**	**175.7±9.9**	**166.7±12**	**123.0±32**	**164.3±16**
**Standing**								
	**APB(10)**	**ADM(10)**	**BIC(10)**	**TRI(9)**	**FCR(6)**	**ECR(10)**	**AD(8)**	**PD(9)**
**Onset**	**134.7±8.5**	**130.2±17.0**	**132.9±7.8**	**119.6±3.2**	**117.6±11**	**130.7±4.4**	**121.1±12**	**122.5±16**
**Offset**	**198.9±17**	**170.8±21.1**	**163.8±9.6**	**149.7±14**	**154.7±14**	**181.0±8.6**	**160.5±19**	**151.3±13**
**Wobble**								
	**APB(10)**	**ADM(10)**	**BIC(10)**	**TRI(10)**	**FCR(9)**	**ECR(10)**	**AD(8)**	**PD(9)**
**Onset**	**130.4±11**	**153.6±11.8**	**140.6±13**	**119.2±5**	**106.8±11**	**127.9±3.9**	**142.7±14**	**122.6±15.7**
**Offset**	**182.8±19**	**181.2±9.7**	**175.8±15**	**149.8±5**	**147.6±8**	**155.9±13**	**170.5±22**	**158.3±14.8**

Reflex prevalence is denoted within parentheses per condition and muscle. Latencies are presented as an average ± standard error of the mean in milliseconds.

### Change in Inhibition/ Excitation

**Fig 3:**
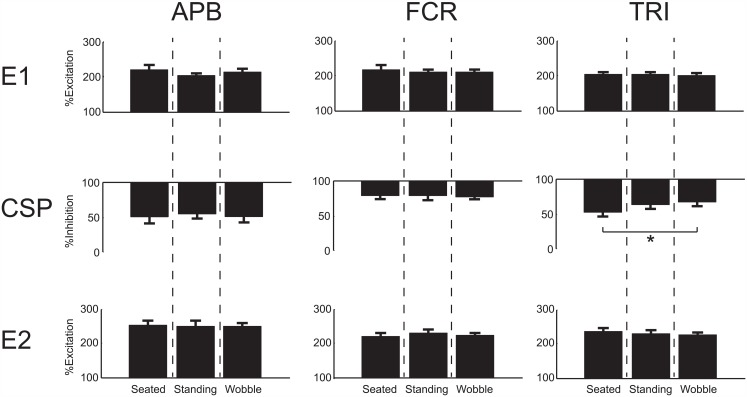
Influence of instability on inhibition/excitation. Group data representing the percentage of excitation (E1, E2) or suppression (CSP) in the EMG for the APB, FCR, and TRI muscles across the 10 subjects (mean ± standard error). A significant increase (*) in the percent of inhibition was observed between the seated and wobble conditions within the TRI muscle (*p* = 0.021). For each of the panels the ‘0’ tick mark represents the baseline EMG value and excitation or suppression is denoted as a percentage of that baseline value. No other significant differences were observed.

There was a significant difference noted in the phase of the reflex response across the three stability conditions for the % of inhibition in TRI (*p* = 0.021). Specifically, the level of CSP inhibition in TRI significantly increased from the seated condition to standing on the wobble board (47.7 ± 7.7% to 33.8 ± 13.2% below baseline, Fig 3). The % of excitation for the two excitatory periods (E1, E2) did not significantly change in TRI with increasing instability. There were no significant effects observed for the % of inhibition or excitation in any of the other muscles, for any of the reflex phases (*p* = 0.39–0.97).

### Duration of Response

**Fig 4:**
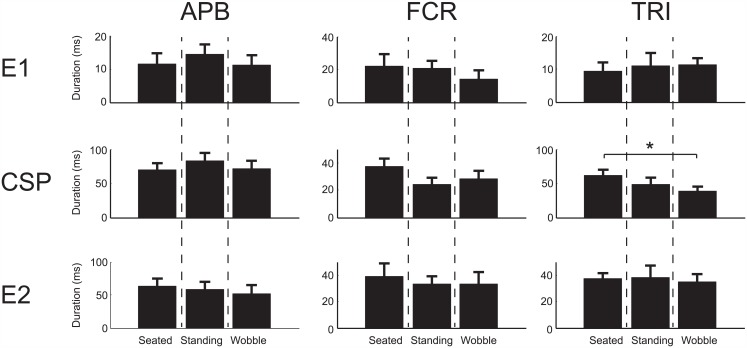
Influence of instability on duration of response. Group data representing the duration of the different responses (E1, CSP, E2) in the EMG for the APB, FCR, and TRI muscles across the 10 subjects (mean ± standard error). There was a significant decrease (*) in the duration of the CSP response was observed between the seated and wobble conditions within the TRI muscle (*p* = 0.034). No other significant differences were observed.

There was a significant difference in the duration of CSP inhibition in TRI, which decreased from the seated condition (62.1 ± 18.2ms) to the wobble board condition (38.1 ± 19.0ms) (*p* = 0.034). The difference in CSP duration is shown in Fig 4. There were no other differences noted in the duration of E1, CSP, or E2 phases in any of the muscles recorded across stability conditions (*p* = 0.086–0.91).

## Discussion

The current study sought to investigate the characteristics of the CSP during a functionally relevant task. The main result demonstrated modulation of the cutaneous silent period (CSP) inhibition in the triceps brachii muscle (TRI) with increasing levels of instability. Secondarily, while present (see Table 2), no significant differences in CSP characteristics were observed in any of the other upper-limb muscles. As a result, when moving from a completely stable position while seated upright in a chair- to standing upright on a wobble board- the amount of cutaneous afferent inhibition of EMG activity in the triceps brachii decreased. This result was coupled with a shorter CSP duration in the triceps brachii with increasing instability. Taken together, these results suggest that the background cutaneous afferent input contributes less to the modulation of ongoing EMG activity in the triceps brachii muscle when performing unstable motor tasks requiring increased use to maintain balance.

Normal motor behavior is a result of complex interactions arising from both cutaneous sensory feedback and descending drive from supraspinal centers. For example, the ability to produce quiet standing is a result of complex processing and coordination between information arriving from the cerebellum, somatosensory cortex, brain stem, and direct peripheral sensory input. Each system provides valuable information that retains the potential to modify human movement, depending on the context. For example, when interacting with an ever-changing environment, it is more efficient to have the descending drive interact with the cutaneous feedback to determine the resultant post-synaptic excitation of the motorneuron pool, thus enabling more precise adjustments with the hand, upper limb, or even the entire body. A physiological mechanism through which such feedback gains can be adjusted in the current study is through presynaptic inhibition of the cutaneous input mediated by the descending drive [20, 21]. Activation of GABAergic interneurons via descending drive has been shown to presynaptically inhibit cutaneous afferents projecting to first-order interneurons (see Fig 1 Rose and Scott, 2003). In this case, the excitatory feedback from cutaneous afferents that achieve, through interneurons, inhibition of the motorneurons is diminished, resulting in less suppression observed in the present study when subjects stand on a wobble board. In turn, less cutaneous inhibition could be concurrent with greater excitation from muscle spindles localized in the triceps brachii or other muscles, adjusting feedback gains from a number of sources to maximize the performance of the wobble board task. This enables the descending drive to work in an integrated fashion with other sensory organs in order to ensure an optimal performance in a dynamic, unstable environment.

As stated earlier, a critical feature of the CSP response is that it is mutable to the task, which is a fundamental requirement for protecting the upper limb when interacting with dynamic environments [3, 9, 10]. Within the current study, this level of mutability can be explained via two different mechanisms. Perhaps the best example of this mutability can be found within feedback from tactile or cutaneous sensory receptors, in particular to protect the limb from noxious stimuli. Specifically, light fingertip touch (< 0.98 N) has the ability to reduce body sway when standing upright [22]. The sensitivity of interactions between the ongoing descending motor commands and the cutaneous feedback can be explained by the optimal feedback control theory [23, 24], where feedback gains (e.g. cutaneous inhibition) are adjusted based on the specific goals of a behavior. Accordingly, the feedback gains are adjusted to maximize performance, which in this case is achieved by maintaining balance on the wobble board and keeping a constant level of background activity in the triceps brachii whether in the CSP, or the two excitatory periods (E1 and E2). Concurrently, this concept lends credence to the theory that there is a modular organization of reflexes. Specifically, the results of this study provide only a significant difference within the triceps brachii. When combining the cutaneous and visual sensory information with the ongoing descending motor command, in the context of the current methodology, a specific response within the upper limb becomes apparent. This is in contrast to other examples of the cutaneous silent period in the hand when all muscles present a clear silent period [5]. Therefore, it may be postulated that a combined effort between the optimal feedback control and modular organization of the reflex are taking place in order to provide the most precise and optimal reflex activity to avoid further insult to the upper limb. Secondly, this mutability can be demonstrated through the persistence of the descending drive within the CSP. For instance, it has been shown that a motor evoked potential (MEP) elicited by transcranial magnetic stimulation (TMS) is still present during the CSP period in hand muscles. The persistence of the motor evoked potential (MEP), though slightly depressed, suggests that the direct cortico-motorneuronal input from the primary motor cortex has the ability to override the inhibitory effects of the cutaneous sensory input to provide an ‘emergency grip’ [15].

The concept of an emergency grip can be best understood if there is a consequence to inhibiting a certain muscle, as would be expected in the triceps brachii if that muscle is providing a semi-rigid link to maintaining stability. Though the triceps brachii is serving the same functional role in all three conditions tested in the present study (seated, standing, on wobble board), the task requirements and demands on the triceps brachii are considerably different. Specifically, if the triceps brachii is briefly inhibited when seated and pushing against the handle, there is no risk of losing balance or risking stability, whereas on the wobble board the consequence can be falling. This could be explained by pairing TMS with cutaneous stimulation as was done in Kofler et al. [15]. That study showed that stimulation of the motor cortical area for the flexor pollicus brevis and first dorsal interosseus muscles in the hand during the CSP period elicited a large MEP suggesting that the descending drive directly synapsing onto the motorneuron pool was weighted more than the inhibitory input from the cutaneous receptors (see Fig 1, Rose and Scott, 2003). However, rather than classifying this as an ‘emergency grip’ as was related to the hand muscles [15], the inability of cutaneous stimulation (even noxious) to abolish the MEP in the triceps brachii muscle suggests that the mechanism is also related to the control of proximal, postural muscles. Thus, while cutaneous feedback aids in normal motor control these studies imply that it is subservient to descending drive from higher centers when needing to increase stability while in an unstable environment.

Regardless of the exact spinal circuitry, which is difficult to ascertain in the present study design, there is a clear change in the weighting of excitatory and inhibitory input to the tricep brachii motorneuron pool, which is specific to the task. Changing from a stable, seated task to an unstable, wobble board task was sufficient to show signs of this altered feedback. However, as was noted earlier, this was limited to the triceps brachii likely due to the tight control of background activity in that muscle during the experiment. Previous reports have demonstrated that the CSP is a mutable response suggesting the ability of the spinal cord to gate cutaneous synaptic input as necessary for a given task [5, 15, 17]. The refinement of specific actions resides in the processing of incoming sensory input from a variety of sources. If it is presumed that the incoming sensory input is then weighted based on immediate need in order to provide the most appropriate response [24], then the most appropriate response, in this case, is maintain a ‘safety margin’ of corticospinal drive then the cutaneous feedback should be weighted less in the wobble board task. It remains important to note, however, that the current study is limited in a variety of ways. First and foremost, the method utilized for the identification of the various components of the CSP response falls under criticism. Specifically, the level of muscle activation and depression should be compared to different techniques employed in other areas to ensure optimality [25, 26]. Along the same lines, the utilized method for reflex identification may lend itself to mistaken muscle activation or depression periods. However, in order to remedy this potential issue, the current study employed the use of visual checks of the data to ensure full reflex activity that included all three components, in line with previously accepted methods allowing for direct comparison with previous and future studies. The data presented in the current study, though largely observational, does provide support for the modulation of the CSP response that allows for functionality in a dynamic environment.
